# The Acute Effects of Upper Extremity Stretching on Throwing Velocity in Baseball Throwers

**DOI:** 10.1155/2013/481490

**Published:** 2013-11-07

**Authors:** Michael Williams, Lanisa Harveson, Jason Melton, Ashley Delobel, Emilio J. Puentedura

**Affiliations:** Department of Physical Therapy, School of Allied Health Sciences, University of Nevada Las Vegas, 4505 Maryland Parkway, P.O. Box 453029, Las Vegas, NV 89154-3029, USA

## Abstract

*Purpose*. To examine the effects of static and proprioceptive neuromuscular facilitation (PNF) stretching of the shoulder internal rotators on throwing velocity. *Subjects*. 27 male throwers (mean age = 25.1 years old, SD = 2.4) with adequate knowledge of demonstrable throwing mechanics. *Study Design*. Randomized crossover trial with repeated measures. *Methods*. Subjects warmed up, threw 10 pitches at their maximum velocity, were randomly assigned to 1 of 3 stretching protocols (static, PNF, or no stretch), and then repeated their 10 pitches. Velocities were recorded after each pitch and average and peak velocities were recorded after each session. *Results*. Data were analyzed using a 3 × 2 repeated measures ANOVA. No significant interaction between stretching and throwing velocity was observed. Main effects for time were not statistically significant. Main effects for the stretching groups were statistically significant. *Discussion*. Results suggest that stretching of the shoulder internal rotators did not significantly affect throwing velocity immediately after stretching. This may be due to the complexity of the throwing task. *Conclusions*. Stretching may be included in a thrower's warm-up without any effects on throwing velocity. Further research should be performed using a population with more throwing experience and skill.

## 1. Introduction

Baseball throwers commonly use stretching to prepare for and improve throwing performance. Performance can be measured in several ways, but one of the most common measures for throwers is throwing velocity. Stretching has long been associated with a typical warm-up for most athletes. Benefits from stretching are thought to include increased range of motion (ROM) and increased flexibility [1]. The neuromuscular mechanisms that may be associated with these effects include reflex inhibition of the Golgi tendon organ and lengthening of the musculotendinous unit [1] which ultimately is believed to increase overall performance in a desired sport or activity, including throwing. However, research has consistently shown that in lower extremity activities that rely on quick bursts of power stretching negatively affects muscle strength and power output immediately after the stretch [2–4]. Researchers have suggested that the observed decrease in power output is most likely due to a decrease in stiffness of the musculotendinous unit. This results in a decreased ability of the muscle to generate force [2].

Less research has been conducted investigating whether these effects would also be observed in the upper extremity. One study, conducted by Haag et al. [5], analyzed the effects of an upper extremity stretching protocol on throwing velocity. Their stretching protocol consisted of 6 static stretches performed in the directions of horizontal adduction, horizontal abduction, external rotation, internal rotation, flexion, and extension. The muscles involved in these motions were chosen because they are active in at least one point of the throwing motion. When compared to the control group, which consisted of a team warm-up without stretching, no difference in throwing velocity was found [5]. Their study focused on increasing the overall ROM of the shoulder. However, based upon results from electromyography (EMG) studies, the shoulder internal rotators are the primary muscles involved in generating power during the throwing motion [6, 7]. Therefore, a more targeted stretch to the shoulder internal rotators may be more appropriate to investigate the effects of stretching on throwing velocity. Haag et al. [5] also limited their protocol to static stretching even though athletes may utilize other stretching techniques. By including other techniques, such as proprioceptive neuromuscular facilitation (PNF), it may be determined if one technique is more effective than another in increasing ROM and flexibility.

Currently, athletes use several techniques of stretching. The most popular of these techniques are static stretching and PNF stretching. Static stretching generally consists of slowly and passively stretching the muscle to a new length. In general, the muscle is stretched to the point where tension limits further movement of the muscle. The stretch is then held for a given period of time. In athletes, a thirty-second stretch is most often used [8, 9]. PNF stretching is popular in sports and focuses on using voluntary muscle contractions to increase ROM by minimizing the resistance of the spinal reflex pathway [10]. The common types of PNF techniques include contract-relax, contract-relax with agonist contraction, hold-relax, and slow-reversal-hold relax [11]. Studies have reported conflicting results about which stretching technique is most effective in increasing joint ROM and muscle flexibility. Puentedura et al. [12] found that there is no difference between PNF and static stretching on hamstring flexibility. However, Funk et al. [13] found that PNF was better at improving acute hamstring flexibility compared to static stretching.

When implementing a stretching program for throwers, it is important to understand the biomechanics of throwing so that the correct musculature can be targeted. The throwing motion can be broken down into three distinct phases: cocking, acceleration, and follow-through. The cocking phase consists of the windup to maximum shoulder external rotation. The acceleration phase begins with maximum shoulder external rotation and continues to the moment of ball release. An increase in maximal shoulder external rotation at this point is correlated with an increase in throwing velocity [14]. The follow-through phase starts with ball release and ends with the termination of the throw [15]. Electromyographic studies have been conducted on the throwing shoulder to determine the muscle activation patterns associated with each phase of throwing. Jobe et al. [6] found that the latissimus dorsi and pectoralis major are most active during the initiation of shoulder internal rotation. Another study by Escamilla and Andrews [7] found that the subscapularis and serratus anterior are also involved. These studies demonstrate that these shoulder internal rotators are primarily responsible for accelerating the arm through the acceleration phase.

Currently, it is not known how stretching the shoulder internal rotators might affect throwing ability. By focusing stretches on these muscles, a change in throwing velocity could be expected due to changes in the musculotendinous unit, similar to that seen in the lower extremity musculature.

The purpose of this study was to examine the effects of static and PNF stretching of the shoulder internal rotators on throwing velocity compared to a typical dynamic warm-up. Based upon our review of the literature, our hypothesis was that stretching (either static or PNF) would result in decreased throwing velocity due to decreased muscle power output associated with presumed changes to the stiffness of the musculotendinous unit.

## 2. Materials and Methods

A repeated measures study was conducted to determine the effects of static and PNF stretching on throwing velocity. Counterbalanced random assignment was used to determine the order of the stretching conditions for each participant. Subjects participated in three separate test days with at least one week in between each session. This was done to wash out any effects of the previous stretching protocol.

### 2.1. Sample

The sample consisted of 27 males between the ages of 20 and 29 years (see Table 1). We were precluded from using professional or collegiate-level baseball players and therefore sought young healthy males with varying levels of baseball throwing experience who were not currently playing at a competitive level. All subjects were right handed; experience of playing team baseball varied from more than 15 years (11.1%); 10–15 years (18.5%); 5–10 years (18.5%); 1–5 years (18.5%); and less than 1 year (33.3%).

Subjects were excluded from the study if they had significant pain or injury in their throwing upper extremity or if they were in a rehabilitation program for a previous injury while the study was being conducted. All subjects provided written informed consent as approved by the University of Nevada Las Vegas Institutional Review Board.

### 2.2. Procedures

**Figure 1:**
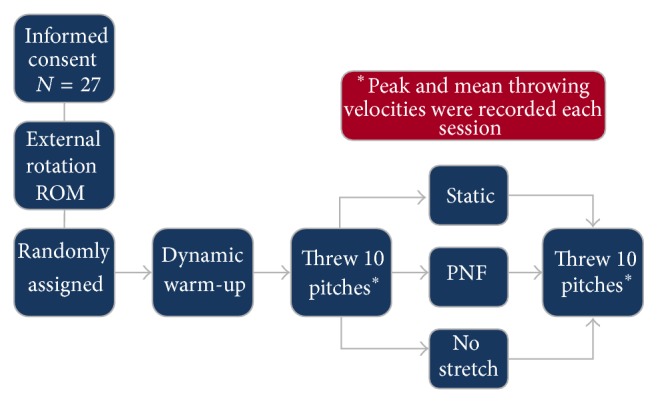
Each session was conducted according to the following flowchart. Subjects were randomly assigned to a stretching condition each session. The procedure was repeated over three different testing days until the subject completed all stretching conditions. Peak and average velocities were recorded each time.

Each subject participated in 3 sessions, each with a different stretching protocol. The order of the protocols was randomly assigned for each subject. We used a counterbalanced randomization such that nine subjects started with each stretching protocol. At the beginning of each protocol, subjects completed a dynamic warm-up that included a light jog of 120 to 180 meters and playing catch (throwing and catching a baseball with another player) for 10 to 15 minutes. The warm-up was concluded when the subjects felt comfortable to participate in throwing at their maximal velocity. Participants were instructed not to include any stretching as part of their warm-up. After their warm-up, each subject was moved to the bullpen of the baseball field, where they threw from the mound, which was 18 meters away from the person catching the ball, and was instructed to throw 10 overhand pitches at their maximum velocity. The subject then received one of the three stretching protocols. Nine of the subjects began their sessions with static stretching, 9 began with PNF stretching, and the final 9 began with the control (no stretch) condition. Ten more pitches thrown at maximum velocity followed the stretching protocols. Velocity was measured for each pitch. Average and peak velocities of the 10 pitches were calculated and used for comparison between the different stretching protocols (see Figure 1).

Stretching protocols focused on stretching the internal rotator muscle group. Based on prior EMG studies, the muscles included were the pectoralis major, latissimus dorsi, and subscapularis [6, 7]. Although the serratus anterior was also shown to be active during the acceleration phase, stretches specific to the muscle were not included due to its primary role as a scapular stabilizer rather than an internal rotator. The pectoralis major muscle was stretched with the subject lying prone on the treatment table, with the throwing shoulder abducted to 90 degrees and externally rotated with the elbow bent to 90 degrees. The examiner stood on the side being stretched and asked the subject to lift his arm off the table towards the sky while the examiner assisted in reaching end range of motion.

**Figure 2:**
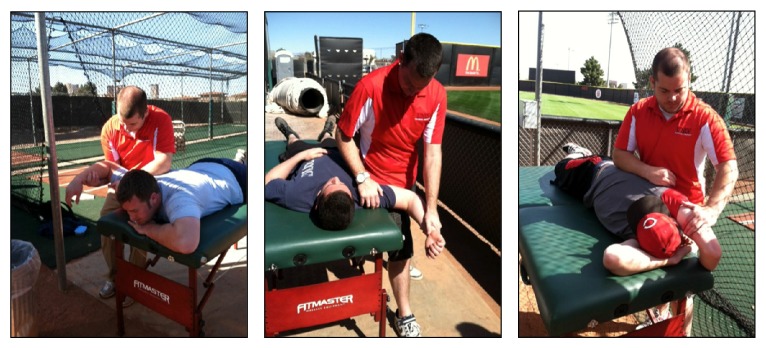
Demonstration of each stretch included in the stretching protocol. Left to right: pectoralis major stretch, subscapularis stretch, and latissimus dorsi stretch.

To ensure the quality of the stretch, the examiner made sure that the subject's sternum remained on the table and the forearm remained horizontal. The latissimus dorsi muscle was stretched in a side-lying position on the side opposite to the throwing shoulder, with his shoulder abducted behind the head with the elbow bent. The knees were bent for comfort and stability. The examiner stood behind the subject and placed one hand on the hip and one on the elbow while pushing the elbow into the table. The subscapularis muscle was stretched with the subject lying supine, the throwing shoulder abducted to 90 degrees, and the elbow flexed to 90 degrees with the shoulder externally rotated as far as possible (see Figure 2). These stretches were adapted from McAtee and Charland [16].

In the static stretching protocol, the stretches were held for 30 seconds at end range and repeated three times with 20 seconds of rest in between each stretch. The PNF stretching protocol utilized the contract-relax technique in which the subject was stretched to end range for 5 seconds, contracted against the examiner for 5 seconds, and stretched to end range again for 20 seconds. The PNF stretches were conducted 3 times with 20 seconds in between each stretch. Total stretching time on each day was about 8 minutes. The same examiner performed the stretching protocols each time. The control protocol involved no stretching with the subject sitting on the stretching table for 8 minutes before throwing again.

### 2.3. Instrumentation

Velocity was measured using a Bushnell Velocity Speed Gun (model number 101911). It has been shown to measure the velocity of a baseball from up to 90 feet (27.5 meters) away and ±1.0 mile per hour (1.6 kilometers per hour) [17]. The examiner operating the radar gun was trained on its proper use to ensure accuracy. The radar gun operator was standing behind and 0.3 to 0.6 meters to the right of the person catching the ball.

### 2.4. Data Analysis

All collected data was analyzed using SPSS version 19.0. To analyze the difference in pre-post-intervention between stretch condition on throwing velocity, two 3 (stretch condition: static stretch, PNF, and no stretch) × 2 (time: before and after) repeated measures ANOVA were performed—one for each of the average and peak throwing velocities. The hypothesis of interest was the condition × time interaction. Simple main effects with a Bonferroni corrected alpha would be utilized if an interaction was observed.

## 3. Results

Although no statistical interaction was found for average throwing velocity (*F*(2,50) = 0.534, *P* = 0.589) or peak throwing velocity (*F*(2,50) = 0.058, *P* = 0.944), main effects were analyzed for both (see Table 2). When analyzing average throwing velocity, the main effect of time was not statistically significant (*F*(1,25) = 3.075, *P* = 0.092). However, the main effect for the stretching groups was statistically significant (*F*(2,50) = 5.267, *P* = 0.008), indicating that when the subjects received the control intervention they threw at greater velocities. Similarly, when analyzing peak throwing velocity, the main effect for time was not statistically significant (*F*(1,25) = 0.065, *P* = 0.800), but the main effect for the stretching groups was statistically significant (*F*(2,50) = 4.342, *P* = 0.018), again indicating that when the subjects received no stretching (control) they were able to achieve greater peak velocities.

## 4. Discussion

The results of this study suggest that neither a static nor a PNF stretching protocol of the shoulder internal rotators had a significant effect on throwing velocity in untrained throwers. Because all subjects underwent their stretching protocols in a counterbalanced randomly assigned order, the significant findings of main effect for stretching groups are difficult to explain. Results indicate that subjects tended to throw faster (average and peak velocity) on the days when they were assigned to the control (no stretch) condition and they threw faster both before and after the 8-minute wait. 

This finding is contrary to the hypothesis that a stretching protocol focusing on the internal rotators of the shoulder would have an effect on throwing velocity since the internal rotators are the muscles primarily responsible for power production while throwing. 

 The finding that stretching prior to throwing had no immediate effect on throwing velocity is inconsistent with previous research conducted on lower extremity activities such as jumping and sprinting [2–4]. Lower extremity research has consistently demonstrated a decrease in power output after stretching. The decrease in performance observed in these previous studies has been attributed to the effects of stretching on the musculotendinous unit. Two main theories have been suggested as possible mechanisms for this reduction in performance [11]. The first proposed by Wilson et al. [18] suggests that a stiff musculotendinous unit is able to produce more force than a lengthened musculotendinous unit due to improved contractile component length and rate of shortening. A second proposed theory suggests that stretching can reduce muscle performance due to autogenic or reflex inhibition [19]. Autogenic inhibition is a spinal reflex resulting from stimulation of the Golgi tendon organ due to an increase in muscle tension. Once stimulated, the Golgi tendon organ causes inhibition of homonymous motor neurons. These effects can last for up to one hour [19].

There are fewer studies and conflicting evidence regarding stretching and its effect on upper extremity muscle performance. One study analyzing the effects of static stretching of the biceps brachii found a decrease in torque and mechanomyography during concentric muscle contractions after a static stretch when compared to a control group that did not stretch. Mechanomyography refers to an observable signal from the surface of a contracted muscle [20]. On the other hand, a study performed by Torres et al. [21] found that neither a static nor a dynamic stretching protocol had any effect on a one repetition maximum bench press, an isometric bench press, an overhead medicine ball throw, or a lateral medicine ball throw. However, researchers hypothesized that this was most likely due to the five minutes of rest between application of the stretches and the performance measures. This study also utilized two 15-second stretches during their treatment. It is possible that this amount of stretching was not sufficient to produce the necessary changes in the musculotendinous unit to cause a decrease in force production [21].

Haag et al. [5] conducted a study examining the effects of static stretching on throwing velocity. The stretching protocol performed in their study focused on improving shoulder ROM in all directions rather than targeting the specific musculature responsible for accelerating the upper extremity throughout the throwing motion. Each of their 6 stretches was performed for 30 seconds and stretching was followed by 5–10 minutes of rest, which was designed to replicate the amount of time a pitcher generally has between warming up and the beginning of the game [5]. The results of the Haag et al. [5] study indicated that there was no difference in throwing velocity after static stretching and the researchers concluded that stretching had no significant effects on throwing velocity. They attributed this mostly to the fact that any acute effects of stretching would be washed out by the period of rest between stretching and throwing [5]. Although the Haag et al. [5] study was of sound quality, a stronger study design may have been to focus on stretching the muscles responsible for providing power during a throw and eliminating the rest time after stretching. By doing this, the acute effects of stretching on upper extremity muscle performance during throwing could be better analyzed.

The present study was designed to focus on the acute effects of stretching on throwing velocity by eliminating stretches of an other musculature and by having the subjects throw immediately after being stretched. The rest time in the current study was less than one minute in most cases. Even by focusing the stretching protocol on the shoulder internal rotators and eliminating a rest period, no significant differences were observed in throwing velocity. The results of this study are consistent with the conclusions in Haag et al. [5] and Torres et al. [21] that suggest that stretching before upper extremity activity does not decrease performance in various upper extremity activities. Specifically, in the current study stretching immediately before throwing was not detrimental to the velocity of the throw. Therefore, baseball throwers may include stretching of the shoulder internal rotators without decreasing throwing velocity.

There are several possible explanations for why a decrease in throwing velocity was not observed in this study. First, throwing a baseball relies on complex neuromuscular patterns [22–24]. Current research suggests that skills, which require complex neuromuscular patterns to complete, may not be affected by stretching. This was observed in a study conducted by Young et al. [25] in which they examined the effects of stretching the hip flexors and quadriceps on foot speed when kicking a football in a group of 16 Australian rules football players. Despite the quadriceps and hip flexors being the musculature primarily responsible for accelerating the leg during the kick, there was no statistically significant difference observed in foot speed. The researchers attributed this lack of difference to the complexity of the neuromuscular pattern required to kick a football [25]. Likewise, in the current study, the complex task of throwing a baseball was not affected by stretching the musculature responsible for accelerating the arm throughout the throw.

If neuromuscular changes were to be observed, a stretch of 30 seconds should have been sufficient to produce measureable changes. Previous researchers have most often utilized a 30-second stretch. Research has consistently shown that ROM benefits are similar for 15-, 30-, 45-, and 120-second stretches [26, 27]. Changes in force production have also been observed following stretches of 30 seconds [28–31]. In the current study, three stretches held for 30 seconds each should have been of sufficient duration to produce a change in force production that would have led to a change in velocity. The stretches utilized also should have been intense enough to produce changes in force production. In the majority of studies, the desired intensity of the stretching protocol was to the point of discomfort, just short of pain [2, 5, 8, 32–35]. The current study used this guideline to direct the intensity of the stretches to the shoulder internal rotators. Even though discomfort and pain are subjective measures and vary with each subject and stretching intensity may not have been uniform for all participants, it should have been of sufficient intensity to elicit the desired neuromuscular effects.

### 4.1. Limitations

A third of the subjects who participated in the study had 0–5 years of baseball throwing experience. For a skilled task such as throwing a baseball, this lack of experience may have affected throwing velocity more than a specific stretching protocol would have, due to the effects of motor learning. When first learning or mastering a new motor skill, individuals tend to coactivate many muscles simultaneously. This inefficient contraction improves with practice until only the necessary musculature is utilized [36]. As the task is mastered, performance is improved [36]. It is possible that these motor-learning effects could have masked any effects elicited by the stretching protocols.

The present study was also unable to account for the finding that when subjects were in the control (no stretch) group they tended to throw faster. This was true both before and after the stretching protocol. The study was designed so that each subject participated in each stretching condition. Therefore, the samples were identical. Also, the order of stretching protocols was randomized in a counterbalanced method for each subject and the subjects were not aware of the order of the protocols. As a result, they could not have anticipated which session they would have been a part of the control group. The only exception to this would have been if the subject was scheduled to participate in the control group during the last session. They would have been aware that they had already participated in the two stretching conditions and would have known that they would not be stretched that day. This could have affected their throwing performance. It should be noted, however, that the difference between the control group's throwing velocities and the other conditions was less than 1.0 mph, which falls within the margin of error for the radar gun used in the study [17]. It is also possible then that there really was no significant difference between the throwing velocities of the stretching groups.

## 5. Conclusion

The primary result of this study was that neither a static nor a PNF stretching protocol of the shoulder internal rotators had any significant effect on a thrower's pitching velocity. This finding indicates that throwers can include stretching of the shoulder internal rotators during their warm-ups without any effects on throwing velocity. Further research in this area should focus on a population that performs overhand pitching at an amateur or professional level. This will help to eliminate any effects of motor learning that might influence the results.

## Figures and Tables

**Table 1:** Descriptive information for the study sample.

Number of subjects	27
Avg. age	25.1 years (SD = 2.4)
Avg. height	180.1 centimeters (SD = 5.3)
Avg. weight	86.8 kilograms (SD = 17.9)
Avg. external rotation	99.1 degrees (SD = 12.3)

**Table 2:** Means and standard deviations for average and peak velocities in each testing condition.

Condition	Average velocity	Peak velocity
Pre-static stretch	92.4 (17.49)	97.5 (17.27)
Post-static stretch	90.6 (17.45)	96.1 (16.85)
Pre-PNF stretch	92.2 (17.48)	97.4 (16.82)
Post-PNF stretch	90.8 (17.11)	96.2 (16.32)
Pre-no stretch	93.5 (17.04)	98.8 (17.30)
Post-no stretch	92.4 (16.72)	98.2 (17.09)

Units = kilometers/hour (SD).

## References

[1] Garber C. E., Blissmer B., Deschenes M. R. (2011). Quantity and quality of exercise for developing and maintaining cardiorespiratory, musculoskeletal, and neuromotor fitness in apparently healthy adults: guidance for prescribing exercise. *Medicine and Science in Sports and Exercise*.

[2] Samuel M. N., Holcomb W. R., Guadagnoli M. A., Rubley M. D., Wallmann H. (2008). Acute effects of static and ballistic stretching on measures of strength and power. *Journal of Strength and Conditioning Research*.

[3] Shrier I. (2004). Does stretching improve performance? A systematic and critical review of the literature. *Clinical Journal of Sport Medicine*.

[4] Marek S. M., Cramer J. T., Fincher A. L. (2005). Acute effects of static and proprioceptive neuromuscular facilitation stretching on muscle strength and power output. *Journal of Athletic Training*.

[5] Haag S. J., Wright G. A., Gillette C. M., Greany J. F. (2010). Effects of acute static stretching of the throwing shoulder on pitching performance of national collegiate athletic association division III baseball players. *Journal of Strength and Conditioning Research*.

[6] Jobe F. W., Moynes D. R., Tibone J. E., Perry J. (1984). An EMG analysis of the shoulder in pitching. A second report. *The American Journal of Sports Medicine*.

[7] Escamilla R. F., Andrews J. R. (2009). Shoulder muscle recruitment patterns and related biomechanics during upper extremity sports. *Sports Medicine*.

[8] Bradley P. S., Olsen P. D., Portas M. D. (2007). The effect of static, ballistic, and proprioceptive neuromuscular facilitation stretching on vertical jump performance. *Journal of Strength and Conditioning Research*.

[9] Norris C. (1999). *The Complete Guide to Stretching*.

[10] Anderson B., Burke E. R. (1991). Scientific, medical, and practical aspects of stretching. *Clinics in Sports Medicine*.

[11] Nyland J. (2005). *Clinical Decisions in Therapeutic Exercise: Planning and Implementation*.

[12] Puentedura E. J., Huijbregts P. A., Celeste S. (2011). Immediate effects of quantified hamstring stretching: hold-relax proprioceptive neuromuscular facilitation versus static stretching. *Physical Therapy in Sport*.

[13] Funk D. C., Swank A. M., Mikla B. M., Fagan T. A., Farr B. K. (2003). Impact of prior exercise on hamstring flexibility: a comparison of proprioceptive neuromuscular facilitation and static stretching. *Journal of Strength and Conditioning Research*.

[14] Wang Y. T., Ford H. T., Ford H. T., Shin D. M. (1995). Three-dimensional kinematic analysis of baseball pitching in acceleration phase. *Perceptual and Motor Skills*.

[15] Pappas A. M., Zawacki R. M., Sullivan T. J. (1985). Biomechanics of baseball pitching. A preliminary report. *The American Journal of Sports Medicine*.

[16] McAtee R., Charland J. (2007). *Facilitated Stretching*.

[17] Bushnell Bushnell performance optics. http://www.bushnell.com/all-products/outdoor-technology/velocity-speed-gun.

[18] Wilson G. J., Murphy A. J., Pryor J. F. (1994). Musculotendinous stiffness: its relationship to eccentric, isometric, and concentric performance. *Journal of Applied Physiology*.

[19] Fowles J. R., Sale D. G., Macdougall J. D. (2000). Reduced strength after passive stretch of the human plantarflexors. *Journal of Applied Physiology*.

[20] Evetovich T. K., Nauman N. J., Conley D. S., Todd J. B. (2003). Effect of static stretching of the biceps brachii on torque, electromyography, and mechanomyography during concentric isokinetic muscle actions. *Journal of Strength and Conditioning Research*.

[21] Torres E. M., Kraemer W. J., Vingren J. L. (2008). Effects of stretching on upper-body muscular performance. *Journal of Strength and Conditioning Research*.

[22] Guido J. A., Stemm J. (2007). Reactive neuromuscular training: a multi-level approach to rehabilitation of the unstable shoulder. *North American Journal of Sports Physical Therapy*.

[23] Wilk K. E., Meister K., Fleisig G., Andrews J. R. (2000). Biomechanics of the overhead throwing motion. *Sports Medicine and Arthroscopy Review*.

[24] Digiovine N. M., Jobe F. W., Pink M., Perry J. (1992). An electromyographic analysis of the upper extremity in pitching. *Journal of Shoulder and Elbow Surgery*.

[25] Young W., Clothier P., Otago L., Bruce L., Liddell D. (2004). Acute effects of static stretching on hip flexor and quadriceps flexibility, range of motion and foot speed in kicking a football. *Journal of Science and Medicine in Sport*.

[26] Madding S. W., Wong J. G., Hallum A., Medeiros J. M. (1987). Effect of duration of passive stretch on hip abduction range of motion. *Journal of Orthopaedic and Sports Physical Therapy*.

[27] Bandy W. D., Irion J. M. (1994). The effect of time on static stretch on the flexibility of the hamstring muscles. *Physical Therapy*.

[28] McHugh M. P., Cosgrave C. H. (2010). To stretch or not to stretch: the role of stretching in injury prevention and performance. *Scandinavian Journal of Medicine and Science in Sports*.

[29] McHugh M. P., Nesse M. (2008). Effect of stretching on strength loss and pain after eccentric exercise. *Medicine and Science in Sports and Exercise*.

[30] Cornwell A., Nelson A. G., Sidaway B. (2002). Acute effects of stretching on the neuromechanical properties of the triceps surae muscle complex. *European Journal of Applied Physiology*.

[31] Young W., Elias G., Power J. (2006). Effects of static stretching volume and intensity on plantar flexor explosive force production and range of motion. *Journal of Sports Medicine and Physical Fitness*.

[32] Knudson D. V., Noffal G. J., Bahamonde R. E., Bauer J. A., Blackwell J. R. (2004). Stretching has no effect on tennis serve performance. *Journal of Strength and Conditioning Research*.

[33] Nelson A. G., Driscoll N. M., Landin D. K., Young M. A., Schexnayder I. C. (2005). Acute effects of passive muscle stretching on sprint performance. *Journal of Sports Sciences*.

[34] Behm D. G., Chaouachi A. (2011). A review of the acute effects of static and dynamic stretching on performance. *European Journal of Applied Physiology*.

[35] Sauers E., August A., Snyder A. (2007). Fauls stretching routine produces acute gains in throwing shoulder mobility in collegiate baseball players. *Journal of Sport Rehabilitation*.

[36] Shumway-Cook A., Woollacott M., Shumway-Cook A., Woollacott M. (2012). Physiological basis of motor learning and recovery of function. *Motor Control: Translating Research into Clinical Practice*.

